# Morphological, cytological and metabolic consequences of autopolyploidization in *Hylocereus* (Cactaceae) species

**DOI:** 10.1186/1471-2229-13-173

**Published:** 2013-11-04

**Authors:** Hagai Cohen, Aaron Fait, Noemi Tel-Zur

**Affiliations:** 1 French Associates Institute for Agriculture and Biotechnology of Drylands, The Jacob Blaustein Institutes for Desert Research, Ben-Gurion University of the Negev, Sede-Boqer Campus, Midreshet Ben-Gurion, Beer-Sheva 84990, Israel

**Keywords:** Flow cytometry, Fruit and seed traits, Pollen diameter and viability, Primary and secondary metabolism, Stomata size and density, Synthetic somatic autopolyploids

## Abstract

**Background:**

Genome doubling may have multi-level effects on the morphology, viability and physiology of polyploids compared to diploids. We studied the changes associated with autopolyploidization in two systems of somatic newly induced polyploids, diploid-autotetraploid and triploid-autohexaploid, belonging to the genus *Hylocereus* (Cactaceae). Stomata, fruits, seeds, embryos, and pollen were studied. Fruit pulp and seeds were subjected to metabolite profiling using established gas chromatography-mass spectrometry (GC-MS) and ultra-performance liquid chromatography (UPLC) Q-TOF-MS/MS (time of flight)-protocols.

**Results:**

Autopolyploid lines produced lower numbers of tetrads, larger pollen grains with lower viability, larger stomata with lower density, and smaller fruits with lower seed numbers and decreased seed viability. The abundance of sugars was lower in the fruits and seeds of the two duplicated lines than in their donor lines, accompanied by increased contents of amino acids, tricarboxylic acid (TCA) cycle intermediates, organic acids and flavonoids. Betacyanins, the major fruit pigments in diploid and triploid donors, decreased following genome doubling. Both autopolyploid *Hylocereus* lines thus exhibited unfavorable changes, with the outcome being more dramatic in the autohexaploid than in the autotetraploid line.

**Conclusion:**

Induced autotetraploid and autohexaploid lines exhibited morphological and cytological characteristics that differed from those of their donor plants and that were accompanied by significant metabolic alterations. It is suggested that a developmental arrest occurs in the fruits of the autohexaploid line, since their pericarp shows a greater abundance of acids and of reduced sugars. We conclude that genome doubling does not necessarily confer a fitness advantage and that the extent of alterations induced by autopolyploidization depends on the genetic background of the donor genotype.

## Background

Genome duplication is one of the main mechanisms by which additional numbers of gene copies are acquired, thereby introducing genetic and evolutionary novelty into organisms [1,2]. The lineages of most flowering plants reflect one or more rounds of ancient polyploidy [3,4]. Polyploidy is, therefore, considered to be a mechanism of adaptation that has played a significant role in plant speciation [5-7]. For almost a century, plant researchers have expressed interest in the mechanisms by which chromosome sets originated to form a polyploid, with focus on the consequences of such processes in both the short and the long terms [8,9]. Studies of natural and newly formed allopolyploids of *Arabidopsis*, *Brassica*, *Glycine*, *Gossypium*, *Tragopogon* and *Triticum* have provided important insights into the genomic and genetic consequences of allopolyploidization [10-18]. Since such polyploids have two different genomes, it is possible that the homoeologous chromosomes – and hence hybridization – rather than polyploidization per se may account for the consequent morphological, physiological and genomic modifications [19]. Autopolyploids that arise within a single species and carry homologous chromosomes have received less scientific attention than allopolyploids, probably because their morphology is often similar to that of their diploid progenitors, and they may therefore have escaped visible identification [5,20-23]. Indeed, the effects of ploidy per se can be studied accurately only in autopolyploids obtained following somatic chromosome doubling – differ from the donor plant only in the genome dosage – rather than in hybridization progenies.

Artificial somatic autopolyploidization can be accomplished both *in vivo* and *in vitro* through the use of antimitotic reagents, i.e., metaphase inhibitors [24]. The methods involve mimic natural systems and may be exploited to produce synthetic somatic autopolyploids with improved traits, i.e., larger fruit and flower size, self-compatibility, improved stress tolerance, and increased biomass and content of some secondary metabolites, among others [24]. It has been shown that the responses elicited by artificial polyploidization significantly affect the morphology and physiology of the newly formed autopolyploids [25-27]. Recently, Aversano et al. [28] reported modifications to the methylation pathways in two synthetic autotetraploids, even though morpho-anatomical analysis did not show any clear differences between the duplicated and the diploid donor lines, thereby suggesting that the changes were stochastic. Metabolic alterations that arise from chromosome duplication have been investigated only in a limited number of studies, and those studies have targeted only specific secondary metabolites, e.g., flavonols and alkaloids, and have therefore “missed” the global metabolic changes associated with autopolyploidization. In several species, such as *Chamomilla recutita*, *Petunia* Mitchell, *Salvia miltiorrhiza*, *Artemisia annua*, and *Panax ginseng*, the production of flavonoids and terpenoids per gram of tissue was higher in polyploids than in their diploid counterparts [29-33].

In light of the significance of autopolyploidization in plant evolution and agriculture and of the insufficient research on this phenomenon, we sought to investigate – by exploiting artificial somatic autopolyploids – morphological traits, cytological changes and metabolic alterations associated with genome multiplication. We hypothesized that autopolyploidization will induce cytological, morphological and metabolic changes, and, more specifically, that there will be strong positive correlations between DNA content and morphological traits, such as stomata, pollen and fruit size, and between DNA content and metabolites.

As model plants for our study, we used species of the hemi-epiphyte *Hylocereus* (Berger) Britton and Rose (Cactaceae). Being plants with Crassulacean acid metabolism, these species are exceptionally tolerant to extreme drought, a significant attribute that has led to their development as exotic fruit crops in dryland agriculture [34]. Axillary vegetative buds of two donor plants, the diploid (2*n* = 2x = 22) species *Hylocereus monacanthus* and a synthetic allotriploid (2*n* = 3x = 33) known as S-75 were treated with an antimitotic reagent, resulting in the production of artificial autopolyploids of 4*x* and 6*x*, respectively, as reported previously [35].

The two duplicated lines and their corresponding donors were subjected to cytological and morphological studies. In addition, changes in the central metabolism and targeted secondary compounds of fruit pulp and seeds were studied by gas chromatography (GC) mass spectrometry (MS) and ultra-performance liquid chromatography (UPLC) Q-TOF-MS/MS (time of flight)-based analyses. The results are discussed within the context of induced somatic autopolyploidization and the genetic and breeding value of the resulting lines.

## Results

### Flow cytometric analysis

Two artificial autopolyploids [4*x* (designated D-27) and 6*x* (designated D-2.3)] were generated from the diploid *H. monacanthus* and the allotriploid S-75, respectively. To verify the success of genome duplication in both autopolyploid lines, we quantified 2C-DNA content using the flow cytometric analysis.

An increase in fluorescence intensity, showing a doubling or close to doubling, in total 2C-DNA was observed in both autopolyploid lines vis-à-vis their respective control lines (Table 1 and Additional file 1), i.e., 4.2 ± 0.1 pg/2C for the diploid *H. monacanthus* vs. 8.0 ± 0.4 pg/2C for the autotetraploid line D-27, and 5.9 ± 0.3 pg/2C for the allotriploid S-75 vs. 13.7 ± 0.1 pg/2C, for the autohexaploid line D-2.3 (Table 1). These results are in line with previously reported chromosome counts [36], thus verifying the success of genome doubling.

**Table 1:** Cytological, morphological and fruit quality parameters of diploid and allotriploid donors and the induced-autopolyploid lines

	***Hylocereus***	**Autotetraploid**	**Allotriploid**	**Autohexaploid**
**Parameter**	***monacanthus***	**D-27**	**S-75**	**D-2.3**
**Cytological parameters**				
Estimated ploidy level	2n	4n	3n	6n
Genome size (pg ±SE)	4.2 ±0.1	8.0 ±0.4*	5.9 ±0.3	13.7 ±0.1*
Stomata diameter (µm ±SE)	53.1 ±0.2	56.0 ±0.2*	63.7 ±0.3	69.0 ±0.3*
Stomata density (±SE)	13.6 ±0.3	12.6 ±0.3*	9.4 ±0.2	8.1 ±0.2*
Number of ovules (±SE)	5036 ±159	3408 ±185*	3530 ±107	3472 ±187
Pollen grain size (µm ±SE)	84.9 ±0.2	107.9 ±0.4*	97.6 ±0.6	122.7 ±0.5*
Pollen stainability (% ±SE)	97.1 ±0.2	86.6 ±1.4*	11.1 ±0.7	52.2 ±4.8*
Tetrads (% ±SE)	99.8 ±0.2	78.3 ±2.4*	74.6 ±0.4	30.3 ±3.9*
Polyads (% ±SE)	0.0 ±0.0	18.9 ±2.2*	9.0 ±2.3	61.1 ±2.7*
Triads (% ±SE)	0.0 ±0.0	2.8 ±0.7*	16.0 ±2.1	6.5 ±1.1*
Dyads (% ±SE)	0.2 ±0.2	0.0 ±0.2	0.3 ±0.2	0.7 ±0.2
Monads (% ±SE)	0.0 ±0.0	0.0 ±0.2	0.1 ±0.1	1.4 ±0.5*
**Morphological parameters**				
Fruit weight (gr ±SE)	333 ±17	267 ±18*	195 ±11	89 ±14*
Peel weight (gr ±SE)	158 ±9	138 ±9	77 ±4	47 ±5*
Flesh weight (gr ±SE)	175 ±10	129 ±10*	118 ±8	42 ±9*
Flesh/Peel ratio (±SE)	1.14 ±0.05	0.95 ±0.08	1.53 ±0.06	0.85 ±0.11*
Black-coat seed number (±SE)	3263 ±201	2190 ±258*	1681 ±113	295 ±37*
Brown-coat seed number (±SE)	0	0	569 ±79	371 ±71
Black-coat seed weight (mg ±SE)	2.57 ±0.02	2.92 ±0.04*	3.86 ±0.06	2.29 ±0.05*
Seed germination (% ±SE)	89 ±1.4	81 ±2.3*	50 ±2.7	30 ±2.5*
**Fruit quality parameters**				
pH (±SE)	3.59 ±0.05	3.33 ±0.06	3.36 ±0.06	2.72 ±0.04*
TSS (% ±SE)	14.1 ±0.31	13.8 ±0.10	16.2 ±0.30	13.6 ±0.30*
Water content (% ±SE)	83.51 ±0.6	84.12 ±0.3	83.46 ±0.1	84.70 ±0.1

*represent statistical significance differences between each of the induced-autopolyploid line to its respective donor line. Statistical analysis was calculated according to the Student’s *t-test* analysis, *P* ≤ 0.05. TSS, total soluble solids.

### Stomata size and density

**Figure 1:**
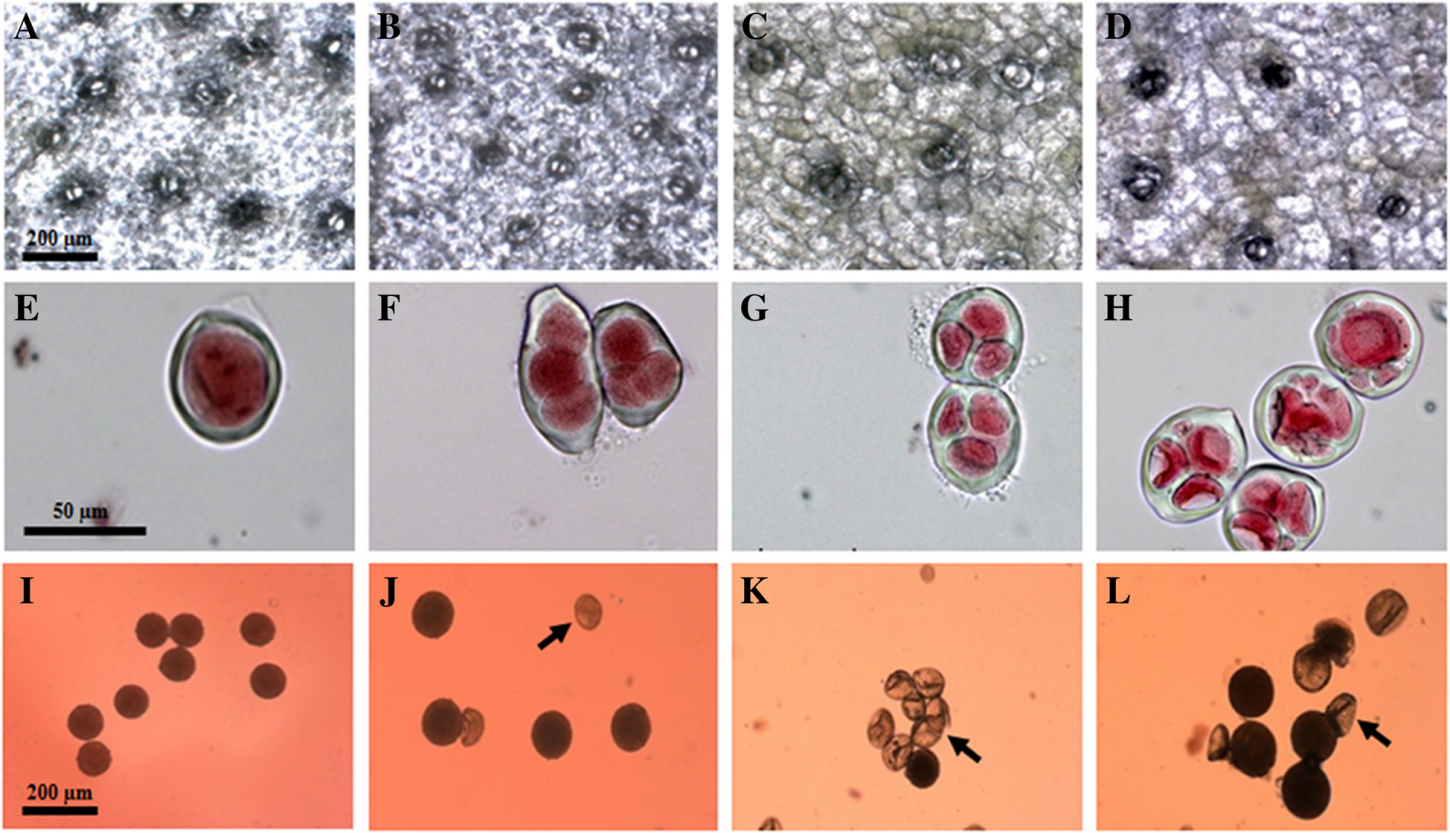
**Stomatal density and size, sporad shapes, and pollen stainability in the donor and autopolyploid lines. A–D**, Stomatal density and size of diploid *H. monacanthus***(A)**, autotetraploid line D-27 **(B)**, allotriploid S-75 **(C)**, and autohexaploid line D-2.3 **(D)**. **E–H**, Sporad shapes in the autohexaploid line D-2.3, monad **(E)**, triads **(F)**, tetrads **(G)**, and polyads **(H)**. **I–L**, Pollen stainability of diploid *H. monacanthus***(I)**, autotetraploid line D-27 **(J)**, allotriploid S-75 **(K)**, and autohexaploid line D-2.3 **(L)**. Arrow indicates an aborted pollen grain.

In accordance with our hypothesis, correlations were observed between DNA content and stomata size and density (Table 1). More specifically, a significant increase in stomata size accompanied by a significant decrease in the density of the stomata was associated with an increase in ploidy level. Average stomata sizes of 53.1, 56.0, 63.7 and 69 μm, and respective densities of 13.6, 12.6, 9.4 and 8.1 stomata per mm^2^, were observed for the donor diploid, the autotetraploid D-27, the control allotriploid S-75 and the autohexaploid D-2.3, respectively (Table 1 and Figure 1A–D).

### Cytological observations

Autopolyploidization did indeed induce cytological changes, as manifested by a marked reduction in the frequency of normal tetrad formation. The frequency of tetrads in the diploid *H. monacanthus* was almost 100%, suggesting normal meiotic division (Table 1). However, for the autotetraploid D-27, the allotriploid S-75, and the autohexaploid D-2.3, there was a decrease in the frequency of tetrads, with the values falling to 78.3, 74.6 and 30.2%, respectively (Table 1). Polyads and triads were observed in the autotetraploid D-27, while polyads, triads, dyads and monads were observed in the allotriploid S-75 and the autohexaploid line D-2.3 (Table 1 and Figure1E–H).

### Pollen grain diameter and stainability

Similarly, correlations were observed between DNA content and pollen grain diameter. The pollen grains of both autopolyploid lines were about 25% larger than those of the donor lines (Table 1 and Figure1I–L). Likewise, pollen stainability was associated with ploidy level, but the effect was not the same in the two autopolyploids. While a small decrease in stainability was observed from the diploid *H. monacanthus* (97.1%) to the autotetraploid D-27 (86.6%), an increase in stainability, meaning recovery of viability, was observed from the allotriploid S-75 (11%) to the autohexaploid D-2.3 (52%) (Table 1).

### Ovule number, fruit and seed traits, and seed germination

**Figure 2:**
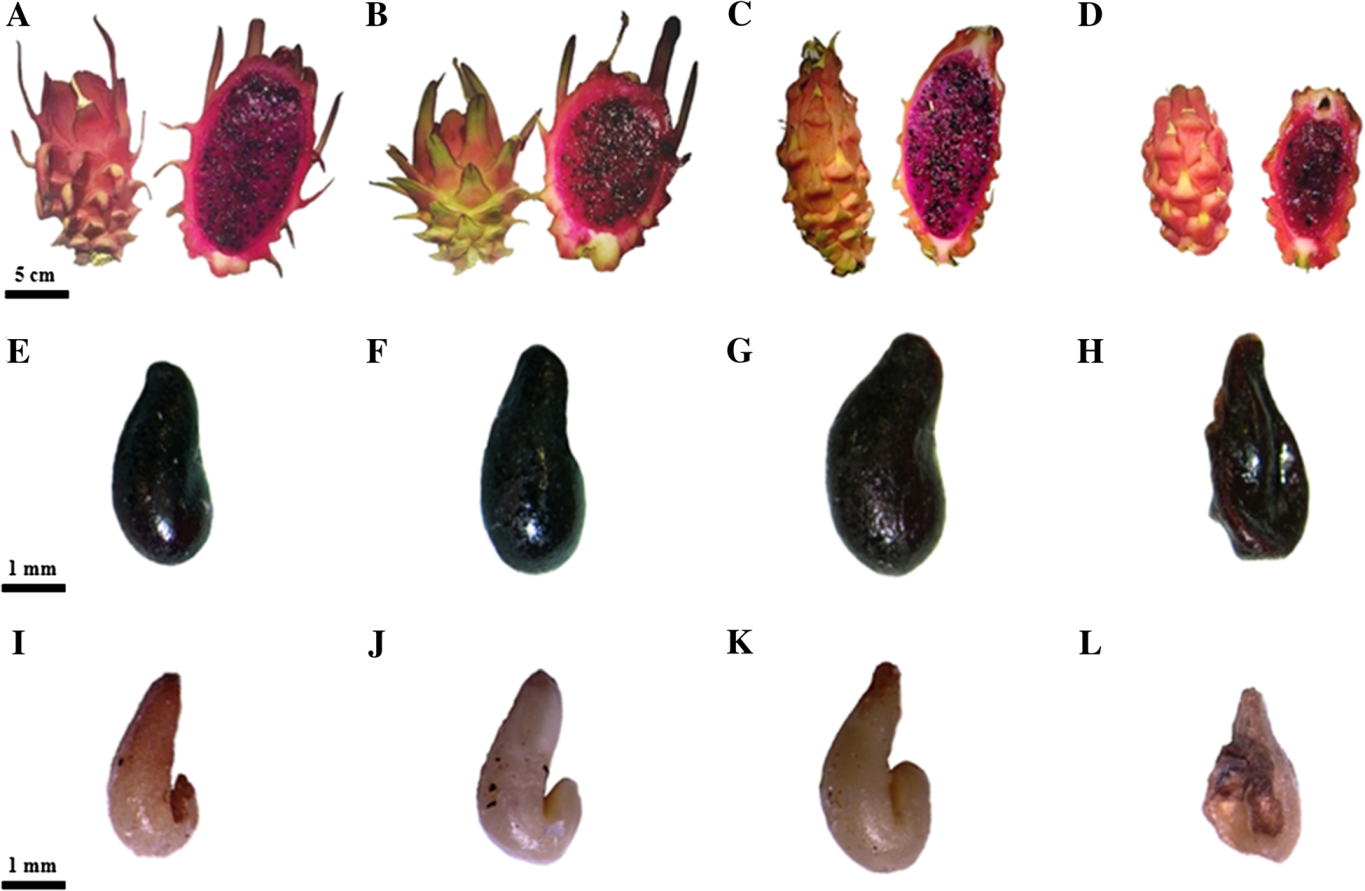
**Morphology of fruit, seed, and embryo tissues in the donor and autopolyploid lines. A–D**, Mature fruits of diploid *H. monacanthus***(A)**, autotetraploid line D-27 **(B)**, allotriploid S-75 **(C)**, and autohexaploid line D-2.3 **(D)**. **E–H**, Black seed separated from the pulp of diploid *H. monacanthus***(E)**, autotetraploid line D-27 **(F)**, allotriploid S-75 **(G)**, and autohexaploid line D-2.3 **(H)**. **I–L**, Embryo removed from black coat seeds of diploid *H. monacanthus***(I)**, autotetraploid line D-27 **(J)**, allotriploid S-75 **(K)**, and autohexaploid line D-2.3 **(L)**.

An association between autopolyploidization and ovule number was observed only in the autotetraploid D-27. An average number of 5,036 ovules per ovary were counted in the diploid *H. monacanthus*, with a significant reduction to 3,048 ovules per ovary being observed in the autotetraploid D-27 (Table 1). However, the differences in observed ovule number between the allotriploid S-75 and the autohexaploid D-2.3 were not statistically significant (Table 1). Alterations in fruit weight, seed weight and number, and germination rate were observed in both autopolyploid lines in comparison with their respective donor lines (Table 1 and Figure 2), indicating that genome doubling had significant effects on these tissues. Fruit weight in both autopolyploid lines was lower than that in their respective donor lines, with the reduction in weight being far more marked in the autohexaploid D-2.3 than in the autotetraploid D-27 (53% and 19%, respectively). In both autopolyploid lines, the reduction in weight was due to the reduction in the pulp weight rather than in the peel weight. These results were confirmed by the lower pulp/peel ratio calculated for both autopolyploid lines (Table 1 and Figure 2A–D).

Total seed number was significantly lower in both autopolyploid lines, i.e., 33% and 70% reduction was observed in the autotetraploid and autohexaploid, respectively (Table 1). Non-viable brown-coat seeds were also observed in both the allotriploid S-75 and the autohexaploid D-2.3. There were also differences in seed weight between the two autopolyploids. While the autotetraploid D-27 set seeds that were significantly heavier (2.92 mg) than those of the diploid donor (2.57 mg), there was a reduction in seed weight in the autohexaploid D-2.3 (2.29 mg) in comparison with the allotriploid donor (3.86 mg) (Table 1 and Figure 2E–H).

Autopolyploidization resulted in a significant reduction in germination rate in both autopolyploids. The highest germination rate was observed for seeds of the diploid donor, reaching almost 90%, whereas those of the autotetraploid D-27 showed a relatively small reduction in seed germination, with the value reaching 81% (Table 1). Despite their normal appearance and shape, the black seeds removed from the allotriploid S-75 had a low rate of germination (50%), and those of the autohexaploid D-2.3 exhibited the lowest germination rate, falling to as low as 30% (Table 1).

The findings for the average seed weights and seed germination rates indicated major differences in seed characteristics between the autopolyploid lines and their respective donors. Thus, the next step was to look for morphological differences in the embryo size and shape. Therefore, the seed coat was opened carefully and the embryos were released. Visually, there was a clear correlation between embryo and seed sizes (Table 1 and Figure 2I–L). Seeds and embryos obtained from fruits belonging to the autotetraploid D-27 were larger than those isolated from the diploid donor *H. monacanthus* (Figure 2I–J). As expected from the correlation with the high average seed weight (3.86 mg), embryos of the allotriploid S-75 were the largest observed (Figure 2K). Seeds and embryos obtained from the autohexaploid D-2.3 were significantly smaller and were characterized by an abnormal shape and patches of necrotic and black tissues (Figure 2L).

### Fruit quality analysis

The consequences of autopolyploidization on fruit quality were evaluated in terms of pulp acidity (pH), total soluble solids (TSS) and water content. A drop in pH and a reduction in TSS levels were observed only in the autohexaploid D-2.3. The water contents were similar (about 84%) in the pulp of the fruits obtained from all four lines, implying that metabolic changes following autopolyploidization are not water-content dependent (Table 1).

### Alterations of primary metabolic profiles following autopolyploidization

To study the effect of autopolyploidization on central metabolism, fruit pulp and seed samples of autopolyploid and donor lines were profiled using an established GC-MS-based protocol [37]. The results were analyzed according to the approach recently suggested by Fernie et al. [38]. Thirty five metabolites were unambiguously annotated for both fruit pulp and seed samples by using an established library (http://gmd.mpimp-golm.mpg.de/), and their relative contents were compared between the autopolyploid line and its respective donor. Statistical significance of the differences was tested using ANOVA (Additional files 2 and 3).

**Figure 3:**
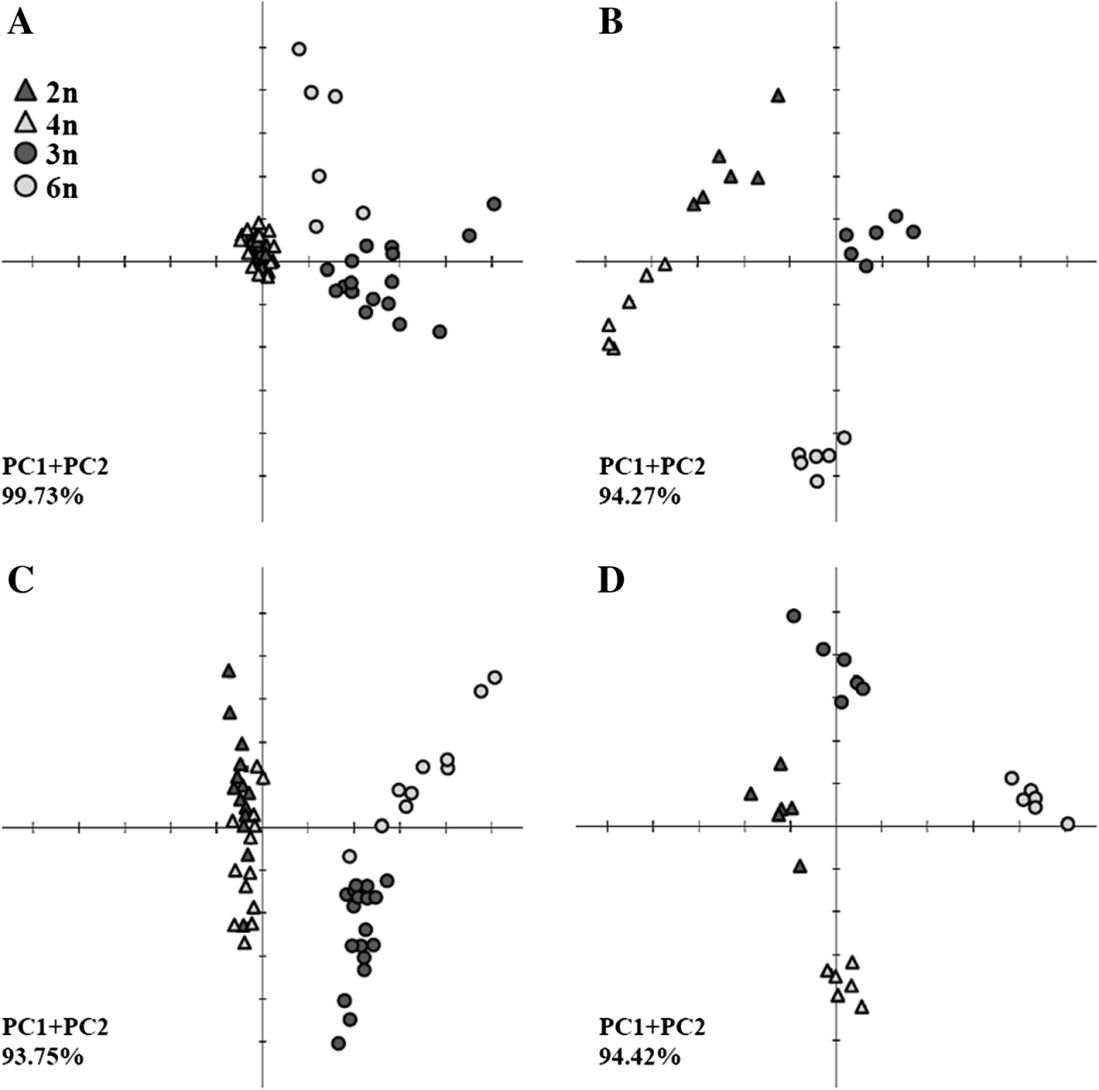
**Principal component analysis (PCA) of GC-MS and UPLC-QTOF-MS/MS metabolite profiling data from fruit pulp and seed tissues of the donors and autopolyploid lines. A–B**, PCA of GC-MS data from fruit pulp **(A)** and seeds **(B)**. **C–D**, PCA of UPLC-QTOF-MS/MS data from fruits pulp **(C)** and seeds **(D)**. The percentage of total variation explained by the first two principal components is shown as PC1 + PC2.

**Figure 4:**
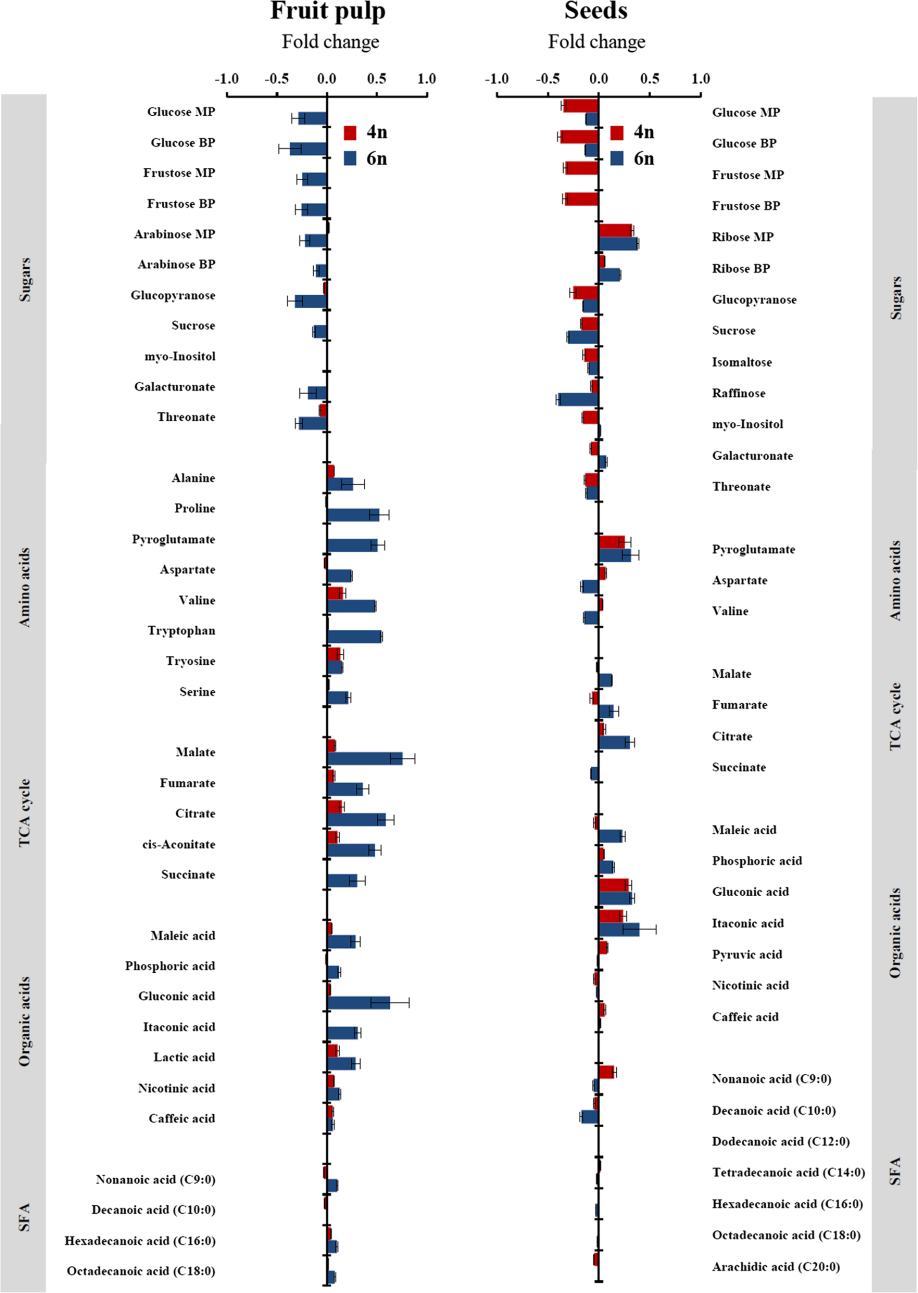
**Changes in the content of primary metabolites of fruits and seeds in response to autopolyploidization.** The bars represent the log_10_-fold change in metabolite content from the induced autopolyploid lines in comparison to their donor lines. The bars facing right (positive) indicate increased content, and the bars facing left (negative) indicate decreased content. Values are presented as the means ± se of thirteen autotetraploid and six autohexaploid independent lines. MP, main product; BP, by product; SFA, saturated fatty acids.

PCA of the metabolite data in the fruit pulp of autopolyploid lines showed extensive changes in the central metabolism in response to the genetic alteration (Figure 3 and Additional file 4). Examination of the principal components showed distinct differences between the autohexaploid and the donor allotriploid samples that were not observed between the autotetraploid and the donor diploid samples (Figure 3A). The effect of autopolyploidization on the levels of individual metabolites of the central metabolism was investigated. As suggested by the PCA, none of the identified metabolites was significantly changed (*P* < 0.01) in the autotetraploid fruit pulp samples. In contrast, significant changes were observed in 22 metabolites of the 35 identified metabolites in the autohexaploid fruit pulp samples. Notably, significant increases (2- to 5.7-fold) in the abundance of most amino acids, tricarboxylic acid cycle (TCA) cycle intermediates, and organic acids were associated with the increase in genome size, with the TCA cycle metabolites showing the most significant changes (Figure 4). The above patterns of change were accompanied by the opposite patterns in the content of most sugars. Six of the eleven identified sugars exhibited 1.8- to 2.4-fold lower concentrations, including glucose and fructose, the two most abundant sugars. The metabolism of saturated fatty acids was not different in the two duplicated lines (Figure 4).

Analysis of the PCA obtained from the seed samples showed clear differences between both duplicated lines and their respective donors (Figure 3B). The significant changes in the metabolic abundance of the seed samples were tested by ANOVA (*P* < 0.01). The metabolite pattern in the seeds was similar to that observed in the fruit pulp samples, i.e., there was significant reduction in the abundance of sugars accompanied by an accumulation of amino acids, TCA cycle intermediates, and organic acids (Figure 4). Of the 35 identified metabolites, 11 showed significant changes in the autotetraploid seed samples, and 15 in the autohexaploid samples. In the autotetraploid seeds, there was a 1.2- to 2.4-fold decrease in the contents of the majority of sugars, while in the autohexaploid seeds fewer sugars were significantly changed, displaying a decrease in their contents up to 2.5-fold (Figure 4). Among the sugars, ribose (main and by-products) showed a marked pattern of change, increasing by up to 2.5-fold in both duplicated lines (Figure 4). Although there were increases in several metabolites of amino acids, TCA cycle products and organic acids in the seeds of both duplicated lines, gluconic acid alone showed a significant (ANOVA) increase (Figure 4). Although more saturated fatty acids were found in the seeds than in the fruit pulp, there were no significant differences in the concentration of these metabolites in the seed samples obtained from the two duplicated lines. This finding implies that induced autopolyploidization did not have a significant effect on the metabolism of fatty acids in the fruit and seed tissues (Figure 4).

To understand how primary metabolic responses in both fruit pulp and seed samples were affected by genome duplication, we examined the coefficient of variation (CV) for each tissue of both autopolyploid lines. Generally, higher CV values characterized the fruit pulp samples of both donors and duplicated lines in comparison to the seed samples, implying a more stable central metabolism in the seed tissues (Additional file 5). The results showed an increase of metabolic variance among samples of fruit pulp from both duplicated lines, with higher values in the autohexaploid samples, implying the impact of genome duplication on the fruit primary metabolome (Additional file 5). In the seed samples, metabolic variance was almost not changed, indicating that the seed central metabolome was less affected by genome duplication (Additional file 5).

### Alterations of secondary metabolic profiles following autopolyploidization

We hypothesized that perturbations induced by the autopolyploidization would lead to direct and indirect modulation of secondary metabolism. Using a UPLC-QTOF-MS/MS-based protocol, we investigated the relative content of the 12 most abundant identified compounds in the fruit pulp and seed extracts (Additional files 6 and 7). These 12 markers were searched against the free chemical database ChemSpider (http://www.chemspider.com/) and quantified on the basis of their relative peak response areas (see Methods). PCA performed for both fruit pulp and seed samples using these 12 most abundant metabolites showed that the autotetraploid and the diploid donor *H. monacanthus* exhibited comparable metabolic profiles, but there were clear differences between the autohexaploid and the donor allotriploid S-75; these findings implied that the effect of autopolyploidization on secondary metabolism was more significant for the fruits produced by the autohexaploid line (Figure 3C). Examination of the PCA of the seed samples revealed marked differences between each duplicated line and its respective donor line (Figure 3D).

**Figure 5:**
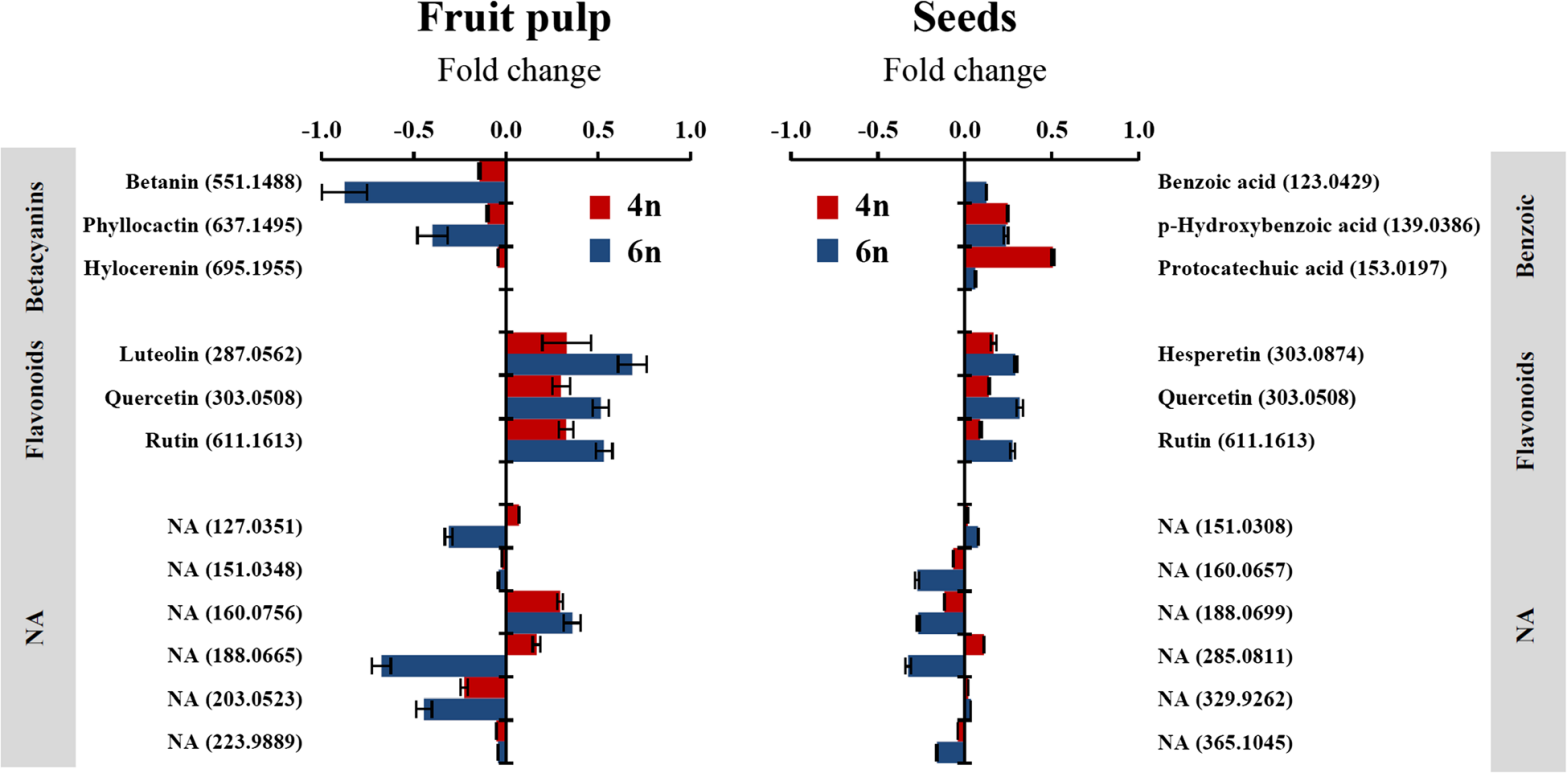
**Changes in the content of secondary metabolites of fruits and seeds in response to autopolyploidization.** The bars represent the log_10_-fold change in metabolite content from the induced-autopolyploid lines in comparison to their donor lines. The bars facing right (positive) indicate increased content, and the bars facing left (negative) indicate decreased content. Values are presented as the means ± se of six autotetraploid and six autohexaploid independent lines. The values in parentheses represent the *m/z* for each metabolite. NA, non-annotated.

The most abundant compounds in the fruit pulp were the three betacyanin pigments, betanin, phyllocactin, and hylocerenin, which were previously identified in *Hylocereus* species [39-43]. These pigments were more abundant in the diploid donor *H. monacanthus* and its respective autotetraploid line than in the allotriploid S-75 and its respective autohexaploid line (Additional file 6). Both duplicated lines exhibited a significant reduction in betanin and phyllocactin, with the decrease being more significant in the autohexaploid than the autotetraploid fruit pulp samples (a 1.4- and 7.5-fold decrease in betanin, and a 1.3- and 2.5-fold decrease in phyllocactin, respectively; Figure 5). The reduction in hylocerenin concentration in the two duplicated lines was not statistically significant (Figure 5).

Three flavonoids, luteolin, quercetin, and rutin, exhibited significant concentration increases in the fruit pulp samples of the duplicated lines, with the accumulation being higher in the autohexaploid line (reaching a 4.9-fold change) than in the autotetraploid line (reaching a 2.1-fold change) (Figure 5).

In addition to the betacyanins and the flavonoids, six abundant but non-identified metabolites were shown to exhibit significant changes in the seed extracts of donor vs. autopolyploid lines. Nevertheless, our efforts to annotate them according to the public libraries were not successful (Additional file 6). For three of these metabolites (annotated as *m/z* 127.0351, 188.0665, and 203.0523), concentrations were significantly decreased, by up to 4.7-fold, while one metabolite (*m/z* 160.0756) displayed a significant 2.3-fold increase. In general, a similar pattern of change for these non-annotated metabolites was shown in the fruit pulp samples, albeit smaller in magnitude (Figure 5).

The abundant metabolites in the seeds were three flavonoids, three benzoic acids and six non-annotated metabolites (three of these non-annotated metabolites were also observed in the fruit pulp samples) (Additional file 7). The flavonoids quercetin and rutin displayed the same patterns of change as those found in the fruit pulp, with significantly increased concentrations of up to 1.5- and 2.1-fold, respectively, in the seed samples of the autotetraploid and autohexaploid lines vis-à-vis their donor lines (Figure 5). Benzoic acid derivatives also increased in both duplicated lines compared to their respective donors (Figure 5). The most significant changes occurred in the seed samples of the autotetraploid line, with *p*-hydroxybenzoic acid and protocatechuic acid increasing by 1.8- and 3.2-fold, respectively (Figure 5). These two metabolites also increased in the seed samples of the autohexaploid line, although to a much smaller extent. Finally, four out of the six non-annotated metabolites (*m/z* 160.0657, 188.0699, 285.0811, and 365.1045) decreased significantly in the seeds of both autopolyploid lines (Figure 5).

## Discussion

Our results indicate that autopolyploidization was accompanied by negative effects, leading to overall disadvantages in plant fitness. These disadvantages included impaired pollen and seed viability, reduced seed germination, smaller fruit size, and lower tetrad number. As expected for such significant morphological and cytological modifications, there were also metabolic changes in the fruits and seeds following genome doubling. In both these tissues, the concentrations of sugars decreased, together with significant increases in the relative abundance of amino acids, TCA cycle intermediates, and organic acids. Targeted analysis of secondary metabolites in the fruit pulp revealed increased abundance of several flavonoids together with decreases in the concentrations of the major betacyanins.

Since plant cell size is correlated with genome size, it may be said that polyploidization is associated with an overall increase in the sizes of cells, tissues and organs [44]. Alterations in cellular architecture may have consequences for regulatory functions, as the cost of supporting the growth of larger genomes [45]. In this work, comparisons between each donor and its autopolyploid revealed – in parallel to the increase in genome size – an increase in stomata size and pollen diameter and a decrease in stomata density. Similar morphological changes have been reported in many other species [46,47] and are associated with an increase in genome size (C-value) but not necessarily with increase in (allo- or auto-) ploidy level [47]. In *Zea mays*, for example, morphological comparison of clones of a ploidy series (1*x*, 2*x*, 3*x* and 4*x*) illustrated the strong effect of genome dosage on the 13 traits studied [48]. However, morphological changes associated with genome dosage are not necessarily the rule. For example, studies on two wild species of *Solanum* did not show consistent differences in terms of stomata size and density between the synthetic autotetraploids and their diploid donors [28], suggesting that the changes in morphological traits are not systematically associated with autopolyploidy. In parallel to the increase in stomata size and pollen diameter, the fact that both our autopolyploid lines produced significantly smaller fruits and lower seed numbers implies that detrimental effects on plant morphology seem to arise from genome doubling. Such morphological changes have been reported in other induced autopolyploids [26,27,49]. In our work, the reduction in fruit size in the autotetraploid line may be attributed to the reduction in the number of ovules, followed by a reduction in the number of black-coat seeds, despite the increase in seed weight. Although the allotriploid S-75 and the autohexaploid D-2.3 showed a similar number of ovules, significant reductions in the number of black-coat seeds and in seed weight were observed in the autohexaploid. The decrease in fruit weight and seed set in the autotetraploid and, more critically, in the autohexaploid, illustrated the disadvantages in plant fitness associated with the increase in ploidy levels in *Hylocereus* species. This conclusion is supported by the fact that the fruits obtained from induced autooctaploid (8*x*) *H. megalanthus* lines (obtained from germinating seeds) ceased developing at an early stage and aborted, resulting in almost zero fruit set [50]. The meaning of our findings is that enlarged cells (such as pollen or stomata) are a consequence of an increase in genome size (C-value) but that these changes are not systematically associated with the enlargement of other plant organs, as fruit weight or number of ovules.

The pairing of two homologous chromosomes, bivalent formation and regular chromosome disjunction have previously been observed in pollen mother cells of the diploid *H. monacanthus*[51], observations that are in line with the formation of normal tetrads (99.8%) and the high pollen stainability (97.1%) reported here. Following genome doubling, a small, but significant, decrease in pollen stainability and in the formation of tetrads alongside a significant increase in the frequency of polyads was observed in the autotetraploid D-27. Even though cytological studies of pollen mother cells and the mechanism(s) of polyad formation are beyond the scope of this work, we can assume that – due to the doubling of the number of chromosomes – meiosis was affected, resulting in the formation of polyads instead of tetrads. Such polyads would be unlikely to form viable pollen grains, thus contributing to the reduction of pollen stainability in the autotetraploid line. Despite the relatively high number of tetrads (74.6%) observed in the allotriploid S-75, pollen stainability was very low (11.1%). Thus, we assumed that most of the products of the tetrads, as well as the triads and the polyads, contain incomplete sets of chromosome complements and are therefore unlikely to yield functional male gametes. The fertility of triploid plants is rarely zero [52], and genome doubling provides new sets of homologous chromosomes, as happened in our autohexaploid line, resulting in partial restoration of fertility (52.2%).

We also observed an overall reduction in the total seed number with an increase in ploidy level. However, the ratios of ovule number/seed number were similar (64-65%) in the diploid, autotetraploid and allotriploid lines, with a value of about 19% in the autohexaploid D-2.3. These findings indicate that the fertility of the female gametes in the allotriploid S-75 was higher than that in its male gametes, with the opposite situation occurring in the autohexaploid D-2.3. Regarding seed germination, lower levels of germination were associated with a higher ploidy level, but the effect was more severe in the autohexaploid D-2.3 (30%), which also showed a very low seed mass, abnormal seed shape and necrotic embryo tissues; these findings once again reflected the detrimental changes at the highest ploidy level. These results are in line with previously published works showing that an increase in seed mass in polyploids is not certainly related to rapid and/or successful germination [53].

While it has been shown that allopolyploidization leads to rapid genetic and epigenetic modifications, resulting in multi-level cellular perturbations [14,16], it has long been believed that autopolyploidy would not involve such alterations, since the duplication event originates from identical homologous genomes [22,54]. The induced-autopolyploid lines may exhibit enhanced production of metabolites, which serve as a useful plant material that can be exploited to attain rapid genetic improvement with respect to the production of secondary metabolites [55]. Several works have, however, shown variable effects of genome dosage on the production of metabolites. The tetraploid *Lycopersicum esculentum* showed a diverse enzymatic response to polyploidization, i.e., malate dehydrogenase, acid invertase and acid phosphatase activity increased, while peroxidase activity decreased and neutral phosphatase and esterase concentrations were not affected (reviewed in [53]). In a metabolic comparison between tetraploid lines generated from the diploid *Cucumis sativus* L., Filipecki et al. [56] concluded that differences in metabolic profiles were tissue-culture responsive and did not correlate directly with the range of genome changes in tetraploids. We note here that while all of the above studies have made a valuable contribution to understanding the effects of gene dosage, these studies were targeted to specific metabolites. In our study, GC-MS-based metabolite profiling showed coordinated changes in response to the increase in genome dosage. Sugars decreased significantly as a result of autopolyploidization, accompanied by a dramatic accumulation of amino and organic acids. These responses were correlated with our fruit quality measurements, which showed that the autohexaploid fruits alone exhibited lower pH and TSS values. It is likely that a developmental arrest is reason for the metabolic changes in the fruits, which manifested as smaller sized fruits. This notion is supported by the developmentally induced changes in the metabolism of other species. For example, Carrari and Fernie [57] observed that during tomato fruit ripening, the levels of sugars increased significantly accompanied by a decrease in most amino acids and TCA cycle intermediates. Increased synthesis of secondary metabolites as a response to abiotic and/or biotic stress is frequently observed in plants. For example, a comparison of the profiles of betacyanins produced by epidermal layers of non-stressed grafted and lightly stressed cactus stems showed higher levels for the stressed stems [58]. In *Beta vulgaris* leaves, wounding and bacterial infiltration was followed by oxidative-burst-induced betacyanin synthesis [59]. Leaf epidermis cells of *Mesembryanthemum crystallinum* irradiated with high light displayed a rapid cell-specific accumulation of flavonol and betacyanin [60].

Fruits of *Hylocereus* species are characterized by unique betacyanin pigments, including the previously identified metabolites betanin, phyllocactin, and hylocerenin [40], that are responsible for the red color of the flesh and that exhibit antioxidant and antiproliferative activities [61,62]. The betacyanins are regulated positively by cytokinins [63,64] and negatively by ABA [65]. In our fruit pulp samples extracted from both duplicated lines, the most significant change in secondary metabolites following autopolyploidization was the decrease of the main betacyanins. This decrease was accompanied by a significant increase in several flavonoids, such as luteolin, rutin and quercetin. Rutin and the products of its conversion to quercitin and downstream flavonoids have been associated with developmental changes and senescence in leaves [66,67]. The above lines of evidence and earlier studies suggest a possible shift from the biosynthesis of betacyanins to flavonols, possibly by the modulation of dihydroflavonol reductase, the first committed enzyme of anthocyanin biosynthesis in the flavonoid pathway, which catalyzes the NADPH-dependent reduction of dihydroflavonols into leucoanthocyanidins [68].

In has long been known that structural and metabolic changes occur during fruit pulp and seed development and that there are mutual interactions between fruit and seed tissues [69]. It is also known that compositional metabolic changes take place during seed development [70,71]. However, no data are available on metabolic changes in seeds following autopolyploidization, and there are no studies comparing the metabolic patterns of seed and fruit pulp tissues following this process. Our results show that the patterns of change in the primary metabolites in the seeds were similar to those observed in the fruits. However, in the seeds, the increases in the abundance of metabolites belonging to the groups of amino acids, TCA cycle intermediates, and organic acids were less marked than those observed in the fruits. These observations, along with the fact that lower CV values characterized the seed samples in comparison to the fruit pulp, suggest a more stable primary metabolic network in the seed, which is less affected by genomic perturbations driven by autopolyploidization.

## Conclusion

Our results suggest that autopolyploidization in *Hylocereus* species led to multi-level negative alterations, which were intimately interrelated. Both duplicated lines were significantly affected by genome doubling and, as a consequence, the meiotic behavior changed, and the morphological traits of fruits, seeds and embryos were all altered, as were the metabolic pathways. Furthermore, metabolite profiling showed a wide range of metabolic changes, which were probably associated with the fruit ripening process and/or cellular stress.

With regard to the evolutionary implications and the breeding potential of new autopolyploids – and in line with previously reported works – we conclude that the response to autopolyploidization – manifested morphologically, cytologically and metabolically – is species specific, depending on the genetic background of the donor genotype rather than on the process of genome doubling.

## Methods

### Plant material and growth conditions

The plant materials used in this study were the diploid *Hylocereus monacanthus* (Lem.) Br. and Rose, 2*n* = 2*x* = 22, accession 89-028; the allotriploid S-75, 2*n* = 3*x* = 33, an interspecific-interploidy cross between the diploid *H. monacanthus* and the tetraploid *Hylocereus megalanthus* [(Schum. ex Vaupel) Moran] Bauer; and their synthetic autopolyploids, i.e., the autotetraploid *H. monacanthus* (named D-27) and the autohexaploid S-75 (named D-2.3). The somatic autopolyploidization process and chromosome counts of lines D-27 and D-2.3 have been reported previously [35]. The two donor plants and their somatic autopolyploids were propagated vegetatively: cuttings were planted in 15-L pots, and all plants were grown under a fertigation regime of 2-L of water per day mixed with 23:7:23 NPK fertilizer at a concentration of 100 ppm. The study was carried out in a greenhouse under 50% of shade located on the Bergmann Campus, Ben-Gurion University of the Negev, Beer-Sheva, Israel during two consecutive fruiting seasons (2009–2010 and 2010–2011).

### Flow cytometric analysis

Genome size was assessed by comparing the nuclear DNA content of the diploid *H. monacanthus* and the allotriploid S-75 with the corresponding autopolyploid lines D-27 and D-2.3, as described in Cohen and Tel-Zur [50]. Each line was analyzed at least four times to verify reproducibility.

### Stomatal size and density

Epidermis layers were separated from fully expanded mature branches and then mounted on a microscope slide with two drops of doubly distilled water. Stomatal density was determined in the diploid *H. monacanthus*, the allotriploid S-75, the autotetraploid D-27, and the autohexaploid D-2.3 by counting the number of stomata per 1 mm^2^ area. For each line, 45 fields were observed from different branches of five plants. Photomicrographs were taken with a Zeiss Axio Imager A1 light microscope, and stomatal size was measured using a Zeiss Axiocam MRc 5 camera, AxioVision program, version 4.6.3.0 SP1.

### Cytological observations

Flower buds were collected from the diploid *H. monacanthus*, the allotriploid S-75, and the autopolyploid lines D-27 and D-2.3 and fixed for 24 h in 3:1 ethanol:glacial acetic acid. The buds were then stored in 70% ethanol at 4°C. The sporads were stained in a drop of 2% acetocarmine, observed under a Zeiss Axio Imager A1 light microscope, and photographed with a Zeiss Axiocam MRc 5 camera, using the AxioVision program, version 4.6.3.0 SP1. About 500–700 sporads from each line were viewed and classified according to structure.

### Pollen grain diameter and stainability

Pollen grains were collected at anthesis from all four plant lines and stained with 2% acetocarmine (acetocarmine was chosen because it gives good results with stored pollen). About 300–500 pollen grains from at least 10 different flowers were measured for each line. Photomicrographs were taken with a Zeiss Axioimager A1 light microscope, Zeiss Axiocam MRc 5 camera, AxioVision program, version 4.6.3.0 SP1.

### Ovule number, fruit and seed traits, and seed germination

Seven floral buds from each of the four lines were collected at anthesis, and the number of ovules per flower bud was counted. Fruits were collected from the two donor lines and the two autopolyploid lines at full maturation and analyzed for morphological traits. Total fruit weight, peel and pulp weight, and total seed number/fruit were measured for at least 25 fruits from each line. Average seed weight was determined using a total of 500 seeds from each line.

A germination assay was performed only on black coated seeds for a total of 100 seeds for each line. Seed germination was studied by placing 20 seeds on wet filter paper in a Petri dish and counting the number of seeds that had germinated after 10 days.

Fruit quality parameters were measured in the juice extracted from 15 fruits from each line. The acidity of the juice was determined by measuring the pH (Eutech Instruments, CyberScan pH 510). TSS was determined with a refractometer (PR-100, Atago, Japan). Water content was determined by separating approximately 15 mg of pulp from the seeds, drying the material in a hybridization oven (Thermo Electron Corporation, Hybaid Shake ’n’ Stack oven) at 70°C, and then reweighing it.

### Extraction and analysis of metabolites with GC-MS and UPLC-QTOF-MS/MS

Fruit pulp (150 μl of pulp separated from the seeds with the use of a strainer) or seed samples (25 mg of dry seeds) were collected into 2-ml tubes, snap frozen in liquid nitrogen, and then stored at -80°C under argon until further analysis. Samples were treated according to an established GC-MS/LC-MS protocol [37,72], i.e., primary metabolite analysis in a GC-MS DSQII (Thermo-Fisher Ltd.) and secondary metabolite analysis in a UPLC-QTOF-MS/MS system equipped with an electrospray ionization (ESI) interface (LC, Waters Acquity UPLC system; MS, Waters Q-TOF Premier) operated under the conditions described by Bing et al. [73]. For the GC-MS data, mass spectral searching was performed utilizing the National Institute of Standards and Technology (NIST, Gaithersburg, MD, USA) algorithm incorporated in the Xcalibur® data system (version 2.0.7) against accessible mass spectral (MS)-retention index (RI) libraries available from the Max-Planck Institute for Plant Physiology, Golm, Germany (http://csbdb.mpimp-golm.mpg.de/csbdb/gmd/msri/gmd_msri.html) and finally normalized to the internal standard ribitol. The raw data obtained by the UPLC-QTOF-MS/MS were recorded by the MassLynx® software (Waters) (version 4.1). The metabolites identified by MassLynx were searched against the chemical database ChemSpider (http://www.chemspider.com/). The quantification of the compounds was based on the relative peak response area of each mass signal after Pareto scaling in the chromatograms and normalized to the internal standard ampicillin [74].

### Statistical analysis

Comparisons were performed between the diploid *H. monacanthus* and the autotetraploid D-27 and between the allotriploid S-75 and the autohexaploid D-2.3. Statistical significance of GC-MS and UPLC-QTOF-MS/MS analyses was calculated using ANOVA (*P* < 0.01), while the Student’s *t*-test (*P* < 0.05) was used to evaluate the statistical significance for all cytological, morphological, and fruit quality analyses, both through the JMP program, version 8.0. PCA was used to visualize the global differences in metabolite profiles between the control and the induced-autopolyploid lines. All PCAs were performed with the free software package TMEV [75] with a default weighted covariance-estimation function. Coefficient of variation (CV) values were calculated on Microsoft Excel by taking the ratio of the standard deviation over the mean for every metabolite individually for either fruit pulp or seed samples from both donors and autopolyploid lines. Thereafter, the resulting CV values were divided into 12 bins of incremental intervals of 0.1, for which the relative frequencies were estimated.

## Abbreviations

GC-MS: Gas chromatography-mass spectrometry; PCA: Principal component analysis; TCA: Tricarboxylic acid cycle; TOF: Time of flight; TSS: Total soluble solids; UPLC: Ultra-performance liquid chromatography; CV: Coefficient of variance.

## Competing interests

The authors declare that they have no competing interests.

## Authors’ contributions

AF, NTZ conceived the study. HC carried out the experiments and data analysis. AF coordinated the metabolic studied. NTZ coordinated the cytological and morphological studies. HC, AF and NTZ wrote the manuscript. All authors read and approved the final manuscript.

## Supplementary Material

Additional file 1 **Flow cytometric analysis of donors and autopolyploids lines.** Plots of (A) diploid *H. monacanthus*, (B) autotetraploid line D-27, (C) allotriploid S-75, and (D) autohexaploid line D-2.3. G0/G1 peak position of the diploid *H. monacanthus* accession 89-028 was compared with the control allotriploid S-75 and the autopolyploid lines D-27 and D-2.3, and ploidy level was estimated. Each line was analyzed at least four times to verify reproducibility. Click here for file

Additional file 2 **Relative content of primary metabolites obtained from fruit pulp GC-MS analysis and (log10) fold change in the autopolyploid lines.** Mass spectral searching utilized the algorithm incorporated in the Xcalibur® data system and finally normalized by the internal standard ribitol. MP = main product, BP = by product. Asterisks represent significant changes in metabolite content (*P* < 0.01) according to ANOVA. Fold change represents the (log10) change in the relative content of each metabolite between the autopolyploid lines and their respective control lines. Click here for file

Additional file 3 **Relative content of primary metabolites obtained from seeds GC-MS analysis and (log10) fold change in the autopolyploid lines.** Mass spectral searching utilized the algorithm incorporated in the Xcalibur® data system and finally normalized by the internal standard ribitol. MP = main product, BP = by product. Asterisks represent significant changes in metabolite content (*P* < 0.01) according to ANOVA. Fold change represents the (log10) change in the relative content of each metabolite between the autopolyploid lines and their respective control lines. Click here for file

Additional file 4 **Metabolite eigenvalues for principal component analysis (PCA).** The metabolites appear below are the seven metabolites with the highest eigenvalues for components one and two, in each of the PCAs performed for GC-MS and UPLC-QTOF-MS/MS data sets, for both fruit pulp and seed tissues. The values in parentheses represent the *m/z* for each metabolite. MP = main product, BP = by product. Click here for file

Additional file 5 **Coefficient of variation (CV) analysis in the fruit pulp and seed samples of autopolyploid lines.** A–B, CV analysis from fruit pulp (A) and seeds (B). Coefficient of variation (CV) values were calculated by taking the ratio of the standard deviation over the mean for every primary metabolite individually for either fruit pulp or seed samples from both autopolyploid lines. Thereafter, the resulting CV values were divided into 12 bins of incremental intervals of 0.1, for which the relative frequencies were estimated. Click here for file

Additional file 6 **Relative content of secondary metabolites obtained from fruit pulp UPLC-QTOF-MS/MS analysis and (log10) fold change in the autopolyploid lines.** Mass spectral searching utilized the algorithm incorporated in the MassLynx® data system and finally normalized by the internal standard ampicillin. Asterisks represent significant changes in metabolite content (*P* < 0.01) according to ANOVA. Fold change represents the (log10) change in the relative content of each metabolite between the autopolyploid lines and their respective control lines. The values in parentheses represent the *m/z* for each metabolite. NA = non-annotated. Click here for file

Additional file 7 **Relative content of secondary metabolites obtained from seeds UPLC-QTOF-MS/MS analysis and (log10) fold change in the autopolyploid lines.** Mass spectral searching utilized the algorithm incorporated in the MassLynx® data system and finally normalized by the internal standard ampicillin. Asterisks represent significant changes in metabolite content (*P* < 0.01) according to ANOVA. Fold change represents the (log10) change in the relative content of each metabolite between the autopolyploid lines and their respective control lines. The values in parentheses represent the *m/z* for each metabolite. NA = non-annotated. Click here for file
