# Supplementary material for: Morphological, cytological and metabolic consequences of autopolyploidization in Hylocereus (Cactaceae) species
Source: BMC Plant Biol. 2013 Nov 4;13:173. doi: 10.1186/1471-2229-13-173 (PMC3831760; doi:10.1186/1471-2229-13-173)

Additional file 1: Flow cytometric analysis of donors and autopolyploids lines. Plots of (A) diploid *H. monacanthus*, (B) autotetraploid line D-27, (C) allotriploid S-75, and (D) autohexaploid line D-2.3. G_0/G_1 peak position of the diploid *H. monacanthus* accession 89-028 was compared with the control allotriploid S-75 and the autopolyploid lines D-27 and D-2.3, and ploidy level was estimated. Each line was analyzed at least four times to verify reproducibility

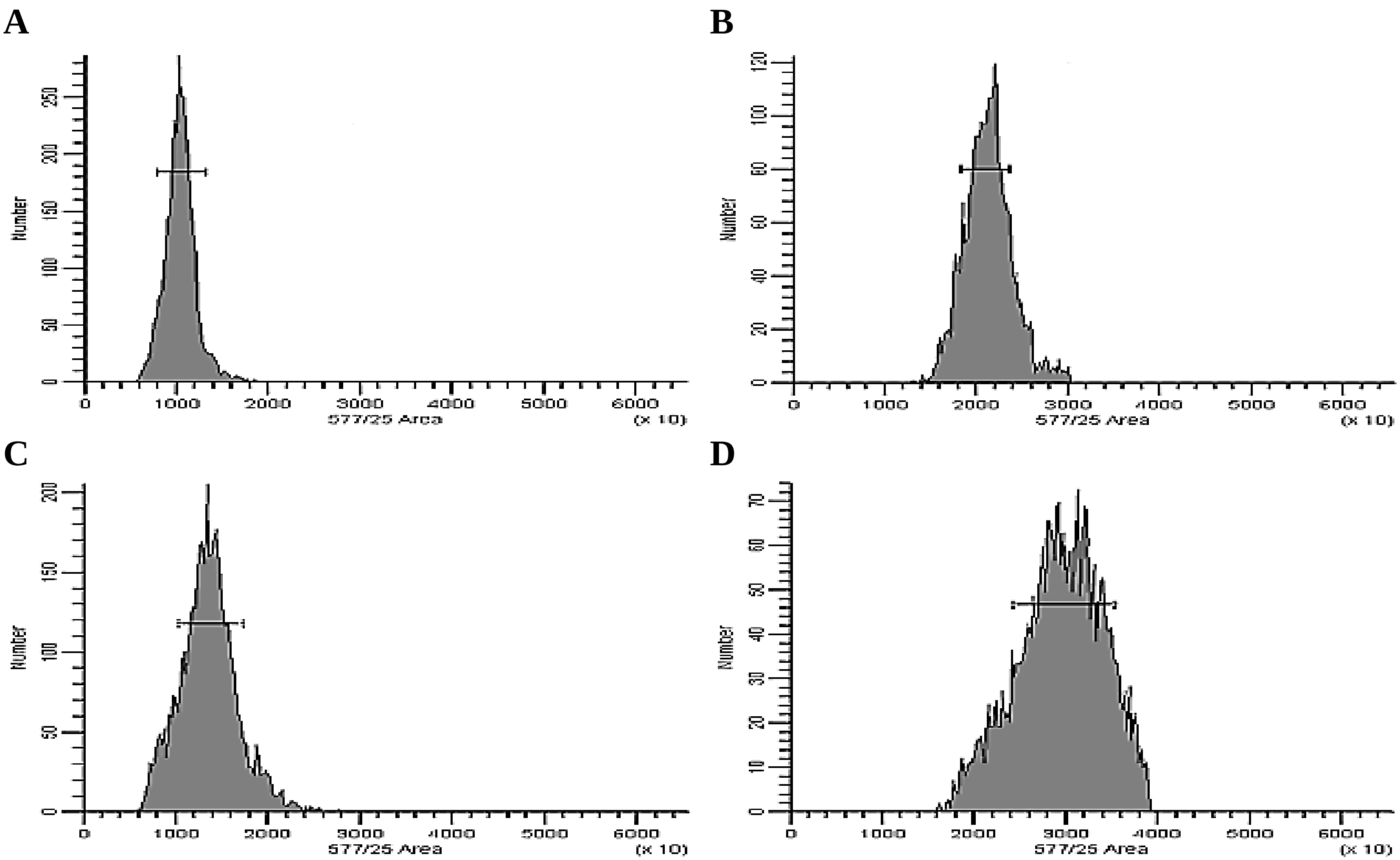

Supplement: Additional file 1 — Flow cytometric analysis of donors and autopolyploids lines. Plots of (A) diploid H. monacanthus, (B) autotetraploid line D-27, (C) allotriploid S-75, and (D) autohexaploid line D-2.3. G0/G1 peak position of the diploid H. monacanthus accession 89-028 was compared with the control allotriploid S-75 and the autopolyploid lines D-27 and D-2.3, and ploidy level was estimated. Each line was analyzed at least four times to verify reproducibility. [file 1471-2229-13-173-S1.pdf]
