# Supplementary material for: Morphological, cytological and metabolic consequences of autopolyploidization in Hylocereus (Cactaceae) species
Source: BMC Plant Biol. 2013 Nov 4;13:173. doi: 10.1186/1471-2229-13-173 (PMC3831760; doi:10.1186/1471-2229-13-173)
Supplement: Additional file 2 — Relative content of primary metabolites obtained from fruit pulp GC-MS analysis and (log10) fold change in the autopolyploid lines. Mass spectral searching utilized the algorithm incorporated in the Xcalibur® data system and finally normalized by the internal standard ribitol. MP = main product, BP = by product. Asterisks represent significant changes in metabolite content (P < 0.01) according to ANOVA. Fold change represents the (log10) change in the relative content of each metabolite between the autopolyploid lines and their respective control lines. [file 1471-2229-13-173-S2.pdf]

Additional file 2: Relative content of primary metabolites obtained from fruit pulp GC-MS analysis and (log_{10}) fold change in the autopolyploid lines. Mass spectral searching utilized the algorithm incorporated in the Xcalibur® data system and finally normalized by the internal standard ribitol. MP = main product, BP = by product. Asterisks represent significant changes in metabolite content ($P < 0.01$) according to ANOVA. Fold change represents the (log_{10}) change in the relative content of each metabolite between the autopolyploid lines and their respective control lines

	Metabolites	2n Average	±SE	4n Average	±SE	Significance	(Log_{10}) 4n fold change	3n Average	±SE	6n Average	±SE	Significance	(Log_{10}) 6n fold change
Sugars	**Glucose MP**	3601.49	188.77	3575.94	383.46		0.00	27951.79	1519.40	14429.41	3388.09	*	-0.29
	**Fructose MP**	1281.93	78.08	1180.14	166.83		0.00	22151.62	1260.36	12547.01	2712.50	*	-0.25
	**Fructose BP**	776.89	47.46	719.29	99.92		0.00	14795.77	884.84	8230.16	2006.52	*	-0.25
	***myo*-Inositol**	621.10	37.09	582.53	61.82		0.00	19140.14	975.76	18915.80	1580.29		-0.01
	**Glucose BP**	492.65	26.65	453.92	53.56		-0.01	6323.09	444.76	2688.23	799.67	*	-0.37
	**Glucopyranose**	12.76	0.59	11.17	1.32		-0.03	126.35	7.47	60.48	14.57	*	-0.32
	**Sucrose**	9.03	0.86	8.42	1.42		0.00	112.51	16.08	83.07	8.84		-0.13
	**Arabinose MP**	1.78	0.18	1.68	0.21		0.02	4.75	0.55	2.86	0.67		-0.22
	**Arabinose BP**	1.09	0.08	0.99	0.09		-0.01	1.30	0.20	1.01	0.28		-0.11
	**Galacturonate**	0.99	0.06	0.93	0.08		-0.01	1.85	0.16	1.19	0.51		-0.19
	**Threonate**	0.86	0.08	0.70	0.05		-0.07	6.94	0.45	3.63	0.46	*	-0.28
Amino acids	**Alanine**	0.03	0.00	0.03	0.00		0.07	0.07	0.02	0.13	0.06		0.26
	**Proline**	3.39	0.59	3.14	0.71		-0.01	128.40	28.06	429.05	80.07	*	0.52
	**Pyroglutamate**	1.28	0.09	1.24	0.15		0.01	21.96	1.80	70.66	9.26	*	0.51
	**Aspartate**	0.38	0.03	0.34	0.05		-0.02	3.95	0.12	6.93	0.23	*	0.24
	**Valine**	0.12	0.02	0.16	0.03		0.16	0.98	0.04	2.99	0.05	*	0.48
	**Tryptophan**	1.37	0.05	1.31	0.03		0.02	1.40	0.05	4.92	0.10	*	0.55
	**Tryosine**	0.08	0.02	0.10	0.02		0.13	0.08	0.00	0.11	0.00	*	0.15
	**Serine**	0.31	0.01	0.30	0.01		0.02	1.16	0.19	1.90	0.25		0.21
TCA cycle	**Malate**	67.02	4.63	76.32	8.47		0.08	47.75	3.68	272.67	43.84	*	0.76
	**Fumarate**	5.61	0.58	6.03	0.88		0.07	1.10	0.13	2.50	0.43	*	0.36
	**Citrate**	3.39	0.32	4.40	0.66		0.15	706.47	70.47	2733.99	384.40	*	0.59
	***cis*-Aconitate**	0.11	0.01	0.12	0.02		0.11	2.32	0.14	7.00	0.89	*	0.48
	**Succinate**	0.21	0.01	0.19	0.01		0.00	0.08	0.00	0.15	0.04	*	0.30
Organic acids	**Maleic acid**	25.01	1.26	26.30	2.20		0.05	44.66	2.21	86.70	13.57	*	0.29
	**Phosphoric acid**	9.81	1.31	9.23	1.48		-0.01	88.44	8.19	116.97	13.70		0.12
	**Gluconic acid**	1.05	0.08	1.07	0.11		0.04	1.32	0.08	5.65	1.71	*	0.63
	**Itaconic acid**	0.17	0.03	0.16	0.02		-0.01	0.68	0.06	1.38	0.14	*	0.31
	**Lactic acid**	0.16	0.01	0.19	0.03		0.11	0.89	0.07	1.74	0.25	*	0.29
	**Nicotinic acid**	0.10	0.00	0.12	0.01		0.07	1.05	0.04	1.41	0.13	*	0.13
	**Caffeic acid**	0.01	0.00	0.01	0.00		0.06	0.38	0.02	0.44	0.09		0.06
Saturated fatty acids	**Nonanoic acid (C9:0)**	0.29	0.03	0.25	0.03		0.02	1.03	0.04	1.30	0.10		0.08
	**Decanoic acid (C10:0)**	0.04	0.00	0.03	0.00		-0.03	0.21	0.02	0.20	0.03		0.10
	**Hexadecanoic acid (C16:0)**	0.50	0.02	0.50	0.03		0.04	2.92	0.10	3.66	0.48		0.10
	**Octadecanoic acid (C18:0)**	1.23	0.04	1.18	0.05		-0.02	4.64	0.23	5.56	0.48		0.00
