# Supplementary material for: Morphological, cytological and metabolic consequences of autopolyploidization in Hylocereus (Cactaceae) species
Source: BMC Plant Biol. 2013 Nov 4;13:173. doi: 10.1186/1471-2229-13-173 (PMC3831760; doi:10.1186/1471-2229-13-173)
Supplement: Additional file 3 — Relative content of primary metabolites obtained from seeds GC-MS analysis and (log10) fold change in the autopolyploid lines. Mass spectral searching utilized the algorithm incorporated in the Xcalibur® data system and finally normalized by the internal standard ribitol. MP = main product, BP = by product. Asterisks represent significant changes in metabolite content (P < 0.01) according to ANOVA. Fold change represents the (log10) change in the relative content of each metabolite between the autopolyploid lines and their respective control lines. [file 1471-2229-13-173-S3.pdf]

Additional file 3: Relative content of primary metabolites obtained from seeds GC-MS analysis and (log_{10}) fold change in the autopolyploid lines. Mass spectral searching utilized the algorithm incorporated in the Xcalibur® data system and finally normalized by the internal standard ribitol. MP = main product, BP = by product. Asterisks represent significant changes in metabolite content ($P < 0.01$) according to ANOVA. Fold change represents the (log_{10}) change in the relative content of each metabolite between the autopolyploid lines and their respective control lines

	Metabolites	2n Average	±SE	4n Average	±SE	Significance	(Log_{10}) 4n fold change	3n Average	±SE	6n Average	±SE	Significance	(Log_{10}) 6n fold change
Sugars	**Glucose MP**	374.20	16.86	168.09	11.59	*	-0.35	351.89	17.78	262.48	8.24	*	-0.13
	**Glucose BP**	28.14	1.41	11.76	0.74	*	-0.38	26.96	1.48	19.88	0.79	*	-0.13
	**Fructose MP**	184.45	9.07	86.58	5.50	*	-0.33	199.28	9.62	193.57	6.12		-0.01
	**Fructose BP**	128.68	6.00	59.97	4.10	*	-0.33	139.19	6.98	136.59	4.65		-0.01
	***myo*-Inositol**	191.45	15.39	133.08	8.22	*	-0.16	503.91	13.86	525.84	10.39	*	0.02
	**Glucopyranose**	0.22	0.01	0.12	0.02	*	-0.25	0.13	0.01	0.09	0.00	*	-0.15
	**Sucrose**	701.12	25.98	471.57	22.13	*	-0.17	685.49	12.64	341.37	8.66	*	-0.30
	**Isomaltose**	0.66	0.04	0.47	0.04	*	-0.14	0.99	0.03	0.78	0.06	*	-0.10
	**Raffinose**	277.80	15.12	234.87	16.23		-0.07	264.57	10.08	105.86	5.26	*	-0.40
	**Ribose MP**	1.57	0.13	3.33	0.17	*	0.33	2.02	0.07	4.87	0.16	*	0.38
	**Ribose BP**	0.39	0.04	0.44	0.03		0.06	0.37	0.01	0.60	0.02	*	0.21
	**Galacturonate**	0.22	0.01	0.19	0.01		-0.08	0.18	0.01	0.22	0.03		0.07
	**Threonate**	1.19	0.06	0.87	0.05	*	-0.14	0.51	0.02	0.38	0.02	*	-0.12
Amino acids	**Pyroglutamate**	3.92	0.69	7.06	1.68		0.26	7.37	0.50	15.26	3.94		0.32
	**Aspartate**	0.49	0.13	0.57	0.06		0.07	2.62	0.12	1.79	0.14	*	-0.16
	**Valine**	5.72	0.29	6.24	0.61		0.04	8.72	0.20	6.27	0.37	*	-0.14
TCA cycle	**Malate**	9.95	0.78	9.53	0.95		-0.02	6.25	0.33	8.44	0.36	*	0.13
	**Fumarate**	1.48	0.44	1.27	0.36		-0.07	0.31	0.07	0.44	0.14		0.15
	**Citrate**	6.37	1.45	7.18	1.89		0.05	39.61	5.29	79.79	11.20	*	0.30
	**Succinate**	0.11	0.01	0.11	0.01		0.00	0.05	0.00	0.04	0.00		-0.07
Organic acids	**Maleic acid**	0.13	0.03	0.12	0.03		-0.04	0.05	0.01	0.08	0.01		0.23
	**Phosphoric acid**	18.16	0.64	20.29	1.13		0.05	17.67	0.66	24.59	1.76		0.14
	**Gluconic acid**	0.51	0.03	0.99	0.11	*	0.29	1.58	0.12	3.36	0.23	*	0.33
	**Itaconic acid**	0.08	0.01	0.14	0.02		0.24	0.19	0.09	0.49	0.20		0.40
	**Pyruvic acid**	0.69	0.07	0.84	0.08		0.08	0.89	0.02	0.85	0.03		-0.02
	**Nicotinic acid**	2.51	0.13	2.26	0.19		-0.04	1.87	0.06	1.78	0.09		-0.02
	**Caffeic acid**	0.03	0.00	0.04	0.01		0.06	0.04	0.00	0.05	0.00		0.01
Saturated fatty acids	**Nonanoic acid (C9:0)**	0.55	0.04	0.78	0.11		0.15	0.48	0.04	0.43	0.05		-0.05
	**Decanoic acid (C10:0)**	0.18	0.04	0.16	0.02		-0.05	0.14	0.02	0.10	0.01		-0.17
	**Dodecanoic acid (C12:0)**	0.22	0.01	0.22	0.01		0.00	0.24	0.01	0.23	0.01		-0.01
	**Tetradecanoic acid (C14:0)**	0.38	0.02	0.39	0.03		0.02	0.34	0.02	0.32	0.01		-0.02
	**Hexadecanoic acid (C16:0)**	9.95	0.54	9.68	0.66		-0.01	8.48	0.25	7.87	0.20		-0.03
	**Octadecanoic acid (C18:0)**	3.25	0.15	3.31	0.25		0.01	2.99	0.09	2.89	0.09		-0.01
	**Arachidic acid (C20:0)**	0.17	0.02	0.15	0.01		-0.05	0.16	0.01	0.16	0.01		0.01
