# Supplementary material for: Morphological, cytological and metabolic consequences of autopolyploidization in Hylocereus (Cactaceae) species
Source: BMC Plant Biol. 2013 Nov 4;13:173. doi: 10.1186/1471-2229-13-173 (PMC3831760; doi:10.1186/1471-2229-13-173)
Supplement: Additional file 4 — Metabolite eigenvalues for principal component analysis (PCA). The metabolites appear below are the seven metabolites with the highest eigenvalues for components one and two, in each of the PCAs performed for GC-MS and UPLC-QTOF-MS/MS data sets, for both fruit pulp and seed tissues. The values in parentheses represent the m/z for each metabolite. MP = main product, BP = by product. [file 1471-2229-13-173-S4.pdf]

Additional file 4: Metabolite eigenvalues for principal component analysis (PCA). The metabolites appear below are the seven metabolites with the highest eigenvalues for components one and two, in each of the PCAs performed for GC-MS and UPLC-QTOF-MS/MS data sets, for both fruit pulp and seed tissues. The values in parentheses represent the *m/z* for each metabolite. MP = main product, BP = by product

**PCA of GC-MS data set obtained from fruit pulp tissues**			
**Metabolite**	**Component 1**	**Metabolite**	**Component 2**
**Glucose MP**	3.665	***myo*-Inositol**	4.918
**Fructose MP**	3.200	**Glucose MP**	2.403
***myo*-Inositol**	3.162	**Fructose MP**	1.156
**Fructose BP**	2.141	**Fructose BP**	1.043
**Glucose BP**	0.874	**Citrate**	0.968
**Citrate**	0.168	**Glucose BP**	0.822
**Proline**	0.027	**Malate**	0.081
**PCA of GC-MS data set obtained from seed tissues**			
**Metabolite**	**Component 1**	**Metabolite**	**Component 2**
***myo*-Inositol**	5.406	**Sucrose**	4.831
**Sucrose**	1.862	**Raffinose**	2.218
**Glucose MP**	1.271	***myo*-Inositol**	1.743
**Fructose MP**	1.211	**Glucose MP**	1.447
**Fructose BP**	0.869	**Citrate**	0.801
**Raffinose**	0.182	**Fructose MP**	0.185
**Citrate**	0.181	**Pyroglutamate**	0.126
**PCA of UPLC-QTOF-MS/MS data set obtained from fruit pulp tissues**			
**Metabolite**	**Component 1**	**Metabolite**	**Component 2**
**NA (160.0756)**	2.379	**NA (188.0665)**	2.329
**Hylocerenin (695.1955)**	1.604	**Hylocerenin (695.1955)**	1.540
**NA (151.0348)**	1.132	**NA (160.0756)**	1.058
**Phylocactin (637.1495)**	0.645	**NA (151.0348)**	0.810
**NA (188.0665)**	0.396	**Phylocactin (637.1495)**	0.688
**NA (127.0351)**	0.353	**NA (127.0351)**	0.405
**NA (551.1488)**	0.322	**NA (551.1488)**	0.403
**PCA of UPLC-QTOF-MS/MS data set obtained from seed tissues**			
**Metabolite**	**Component 1**	**Metabolite**	**Component 2**
**NA (188.0699)**	3.354	**NA (285.0811)**	3.282
**NA (285.0811)**	1.982	**NA (188.0699)**	1.798
**NA (160.0657)**	1.788	**NA (329.9262)**	1.022
**NA (149.0581)**	0.614	**NA (149.0581)**	0.823
**NA (493.2780)**	0.587	**NA (147.0416)**	0.771
**NA (147.0416)**	0.539	**NA (160.0657)**	0.448
**NA (365.1045)**	0.530	**NA (151.0308)**	0.388
