# Supplementary material for: Morphological, cytological and metabolic consequences of autopolyploidization in Hylocereus (Cactaceae) species
Source: BMC Plant Biol. 2013 Nov 4;13:173. doi: 10.1186/1471-2229-13-173 (PMC3831760; doi:10.1186/1471-2229-13-173)

Additional file 5: Coefficient of variation (CV) analysis in the fruit pulp and seed samples of autopolyploid lines. A–B, CV analysis from fruit pulp (A) and seeds (B). Coefficient of variation (CV) values were calculated by taking the ratio of the standard deviation over the mean for every primary metabolite individually for either fruit pulp or seed samples from both autopolyploid lines. Thereafter, the resulting CV values were divided into 12 bins of incremental intervals of 0.1, for which the relative frequencies were estimated

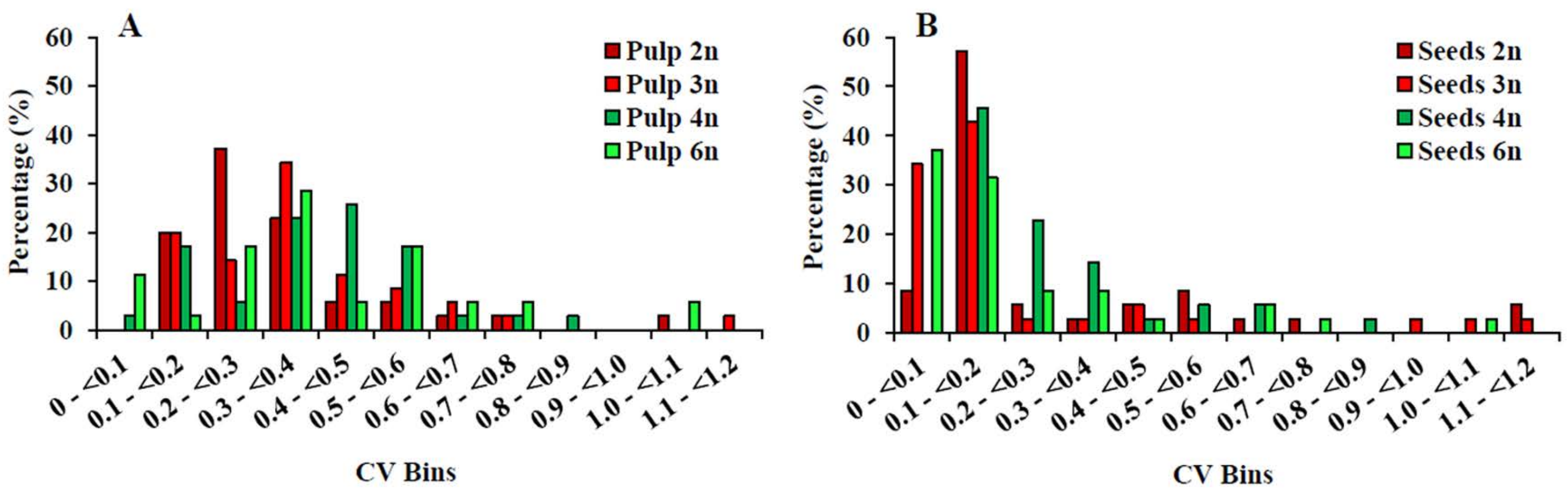

Supplement: Additional file 5 — Coefficient of variation (CV) analysis in the fruit pulp and seed samples of autopolyploid lines. A–B, CV analysis from fruit pulp (A) and seeds (B). Coefficient of variation (CV) values were calculated by taking the ratio of the standard deviation over the mean for every primary metabolite individually for either fruit pulp or seed samples from both autopolyploid lines. Thereafter, the resulting CV values were divided into 12 bins of incremental intervals of 0.1, for which the relative frequencies were estimated. [file 1471-2229-13-173-S5.pdf]
