# Supplementary material for: Morphological, cytological and metabolic consequences of autopolyploidization in Hylocereus (Cactaceae) species
Source: BMC Plant Biol. 2013 Nov 4;13:173. doi: 10.1186/1471-2229-13-173 (PMC3831760; doi:10.1186/1471-2229-13-173)
Supplement: Additional file 6 — Relative content of secondary metabolites obtained from fruit pulp UPLC-QTOF-MS/MS analysis and (log10) fold change in the autopolyploid lines. Mass spectral searching utilized the algorithm incorporated in the MassLynx® data system and finally normalized by the internal standard ampicillin. Asterisks represent significant changes in metabolite content (P < 0.01) according to ANOVA. Fold change represents the (log10) change in the relative content of each metabolite between the autopolyploid lines and their respective control lines. The values in parentheses represent the m/z for each metabolite. NA = non-annotated. [file 1471-2229-13-173-S6.pdf]

Additional file 6: Relative content of secondary metabolites obtained from fruit pulp UPLC-QTOF-MS/MS analysis and (log_{10}) fold change in the autopolyploid lines. Mass spectral searching utilized the algorithm incorporated in the MassLynx® data system and finally normalized by the internal standard ampicillin. Asterisks represent significant changes in metabolite content ($P < 0.01$) according to ANOVA. Fold change represents the (log_{10}) change in the relative content of each metabolite between the autopolyploid lines and their respective control lines. The values in parentheses represent the *m/z* for each metabolite. NA = non-annotated

	**Metabolites**	**2n Average**	**±SE**	**4n Average**	**±SE**	**Significance**	**(Log_{10}) 4n fold change**	**3n Average**	**±SE**	**6n Average**	**±SE**	**Significance**	**(Log_{10}) 6n fold change**
**Betacyanins**	**Betanin (551.1488)**	67.23	4.17	48.37	2.27	*	-0.14	4.30	0.72	0.57	0.13	*	-0.88
	**Phyllocactin (637.1495)**	127.74	5.19	101.07	6.90	*	-0.10	6.10	1.01	2.19	0.48	*	-0.40
	**Hylocerenin (695.1955)**	256.04	12.36	231.20	8.64		-0.04	0.00	0.00	0.00	0.00		0.00
**Flavonoids**	**Luteolin (287.0562)**	2.01	0.18	4.64	1.82		0.33	4.74	0.54	23.03	2.44	*	0.69
	**Quercetin (303.0508)**	3.41	0.40	7.36	1.01	*	0.30	8.17	0.62	26.67	2.17	*	0.51
	**Rutin (611.1613)**	3.14	0.33	6.67	0.81	*	0.33	4.87	0.34	16.60	1.32	*	0.53
**NA**	**NA (127.0351)**	138.95	4.45	163.21	5.92	*	0.07	117.51	2.27	57.52	3.56	*	-0.31
	**NA (151.0348)**	359.51	5.79	341.93	7.91		-0.02	180.06	6.18	164.03	7.59		-0.04
	**NA (160.0756)**	6.91	0.65	13.60	0.76	*	0.29	178.35	9.45	408.79	51.23	*	0.36
	**NA (188.0665)**	137.09	15.74	200.87	24.30		0.17	139.73	11.98	29.52	2.13	*	-0.68
	**NA (203.0523)**	331.55	31.48	196.73	16.63	*	-0.23	60.39	5.69	21.69	2.03	*	-0.44
	**NA (223.9889)**	212.25	5.64	188.20	6.62	*	-0.05	137.06	6.05	124.35	4.95		-0.04
