# Supplementary material for: Morphological, cytological and metabolic consequences of autopolyploidization in Hylocereus (Cactaceae) species
Source: BMC Plant Biol. 2013 Nov 4;13:173. doi: 10.1186/1471-2229-13-173 (PMC3831760; doi:10.1186/1471-2229-13-173)
Supplement: Additional file 7 — Relative content of secondary metabolites obtained from seeds UPLC-QTOF-MS/MS analysis and (log10) fold change in the autopolyploid lines. Mass spectral searching utilized the algorithm incorporated in the MassLynx® data system and finally normalized by the internal standard ampicillin. Asterisks represent significant changes in metabolite content (P < 0.01) according to ANOVA. Fold change represents the (log10) change in the relative content of each metabolite between the autopolyploid lines and their respective control lines. The values in parentheses represent the m/z for each metabolite. NA = non-annotated. [file 1471-2229-13-173-S7.pdf]

Additional file 7: Relative content of secondary metabolites obtained from seeds UPLC-QTOF-MS/MS analysis and (log_{10}) fold change in the autopolyploid lines. Mass spectral searching utilized the algorithm incorporated in the MassLynx® data system and finally normalized by the internal standard ampicillin. Asterisks represent significant changes in metabolite content ($P < 0.01$) according to ANOVA. Fold change represents the (log_{10}) change in the relative content of each metabolite between the autopolyploid lines and their respective control lines. The values in parentheses represent the *m/z* for each metabolite. NA = non-annotated

	Metabolites	2n Average	±SE	4n Average	±SE	Significance	(Log_{10}) 4n fold change	3n Average	±SE	6n Average	±SE	Significance	(Log_{10}) 6n fold change
**Benzoic acids**	**Benzoic acid (123.0429)**	10.04	0.36	9.99	0.38		0.00	16.86	0.31	22.42	0.50	*	0.12
	***p*-Hydrobenzoic acid (139.0386)**	3.95	0.10	6.98	0.11	*	0.25	3.79	0.19	6.54	0.32	*	0.24
	**Protocatechuic acid (153.0197)**	6.06	0.12	19.51	0.35	*	0.51	3.66	0.56	4.22	0.34		0.06
**Flavonoids**	**Hesperetin (303.0874)**	2.79	0.07	3.84	0.10	*	0.17	2.87	0.15	5.61	0.17	*	0.29
	**Quercetin (303.0508)**	1.60	0.07	2.35	0.24	*	0.14	0.55	0.04	1.14	0.06	*	0.32
	**Rutin (611.1613)**	4.91	0.16	6.04	0.39	*	0.09	2.43	0.08	4.58	0.25	*	0.27
**NA**	**NA (151.0308)**	77.23	3.70	80.55	3.12	*	0.02	60.23	1.34	71.74	2.59	*	0.08
	**NA (160.0657)**	242.99	7.77	208.82	2.74	*	-0.07	234.63	7.20	124.94	5.34	*	-0.27
	**NA (188.0699)**	450.09	9.96	342.91	6.68	*	-0.12	425.59	12.86	229.31	7.37	*	-0.27
	**NA (285.0811)**	151.10	5.44	194.66	2.79	*	0.11	55.67	0.85	26.32	1.35	*	-0.33
	**NA (329.9262)**	359.64	9.55	377.38	9.45		0.02	338.31	11.69	366.91	4.55		0.04
	**NA (365.1045)**	80.32	1.48	73.15	1.50	*	-0.04	60.35	1.77	41.71	1.14	*	-0.16
